# Effect of blended and unguided online delivery of mindfulness-based cognitive therapy versus care as usual on distress among cancer patients and survivors: protocol for the three-arm parallel randomized controlled buddy trial

**DOI:** 10.1186/s40359-023-01052-2

**Published:** 2023-01-25

**Authors:** Nasim Badaghi, Mette van Kruijsbergen, Judith Prins, Saskia Kelders, Linda Cillessen, Félix Compen, Rogier Donders, Linda Kwakkenbos, Anne Speckens

**Affiliations:** 1 Department of Psychiatry, Radboud University Medical Center, 966, Postbus 9101, 6500 HB Nijmegen, The Netherlands; 2 Department of Medical Psychology, Radboud University Medical Center, Nijmegen, The Netherlands; 3 Department of Psychology, Health, and Technology, University of Twente, Enschede, The Netherlands; 4 Radboud Institute for Health Evidence, Radboud University Medical Center, Nijmegen, The Netherlands; 5 Optentia Research Unit, North-West University, Potchefstroom, South Africa; 6 Behavioural Science Institute, Clinical Psychology, Radboud University Nijmegen, Nijmegen, The Netherlands

**Keywords:** Internet-based interventions, Mindfulness based cognitive behavioral-therapy, Randomized controlled trial, E-health, Cancer, Psycho-oncology, Unguided therapy, Blended therapy

## Abstract

**Background:**

One third of cancer patients and survivors experience psychological distress. Previous studies have shown that online mindfulness-based cognitive therapy (eMBCT) supports cancer patients and survivors in managing distress. Lack of peer support and asynchronicity during online interventions have been reported as barriers for treatment adherence and can result in higher drop-out rates. Considering this, two new formats of eMBCT were created. The primary objective of the Buddy trial is to evaluate the (cost) effectiveness of blended and unguided eMBCT versus care as usual (CAU) on psychological distress among cancer patients and survivors. Secondary objectives include evaluating effects on other psychological outcomes and investigating working mechanisms and treatment effect moderators.

**Methods:**

The Buddy trial is a parallel three-armed randomized controlled trial. Participants will be randomly assigned to blended therapist-assisted eMBCT, unguided individual eMBCT or CAU. Eligible participants will be Dutch-speaking adult cancer patients or survivors with access to internet. The primary outcome will be psychological distress scores as assessed by the Hospital Anxiety and Depression scale immediately post-treatment. Secondary outcome measures include fear of cancer recurrence (FCRI), fatigue (CIS-F), rumination (RRQ), mindfulness skills (FFMQ), decentering (EQ), self-compassion (SCS-SF), positive mental health (MHCSF), health related quality of life (EQ-5D), and costs associated with psychiatric illness (TiC-P). Outcome measures will be evaluated at baseline, mid-treatment, immediately post-treatment, and three-, six-, and nine-months follow-up. Possible mediators, such as engagement with interventions (TWEETS), and moderators will be also analyzed.

**Discussion:**

There is room to improve eMBCT for cancer patients prior to implementation to ensure adherence and scalability. Blended and unguided eMBCT may reduce psychological distress and improve quality of life and be easily accessible to cancer patients and survivors.

*Trial registration* clinicaltrials.gov, NCT05336916, registered on April 20th, 2022. https://clinicaltrials.gov/ct2/show/NCT05336916.

## Background

It has been estimated that approximately 28.4 million new cancer cases will occur in 2040; a 47% increase from 2020 [27]. While the number of individuals diagnosed with cancer is increasing, so is the survival rate [9]. This means that more people will be living with the consequences of cancer.

Cancer can have a profound impact on people’s physical but also on their mental well-being. It has been estimated that around one in three cancer patients experiences severe psychological distress [32]. Psychological distress has been defined by the National Comprehensive Cancer Network (NCCN) (2012) as “a multifactorial unpleasant emotional experience of a psychological (cognitive, behavioral, emotional), social, and/or spiritual nature that may interfere with the ability to cope effectively with cancer, its physical symptoms, and its treatment”. Psychological distress can manifest in different ways, including anxiety and depression symptoms [22] as well as fear of cancer recurrence [28]. In addition, cancer-related pain and fatigue are common patient-reported problems [6].

Psychological interventions have shown to have positive effects on a variety of cancer-related problems [10]. In the past years, evidence for the effectiveness of mindfulness-based interventions (MBIs), such as Mindfulness-Based Stress Reduction (MBSR) and mindfulness-based cognitive therapy (MBCT), has rapidly increased [5]. Mindfulness is characterized by intentionally paying attention to moment-by-moment experiences in a non-judgmental way [14]. MBSR was originally developed to reduce stress whereas MBCT aimed to prevent relapse in depression [25]. Since their development, however, these interventions have shown beneficial across different domains, and they have been widely implemented for different conditions, including cancer [12].

A recent systematic review and meta-analysis including 29 randomized control trials (RCTs) of MBIs in cancer patients and survivors (N = 3274), revealed that MBIs are effective in reducing psychological distress, anxiety, depression symptoms, fear of cancer recurrence, fatigue, sleep disturbances, and pain in this population, with overall small but robust effects [5]. However, there are known patient-reported barriers to engaging in face-to-face psychological interventions like MBIs, such as investment of time, stigma, fatigue, reluctance to return to the hospital, and indirect costs [3]. Internet-based interventions can offer an alternative, as they are easily accessible and save traveling time.

Although there is little evidence for online mindfulness-based interventions (eMBIs) in the context of psycho-oncology, some preliminary research has shown their potential [4, 19, 33]. Our team has previously conducted the BeMind study, which compared face-to-face group MBCT with therapist-assisted (by email correspondence) individual online mindfulness-based cognitive therapy (eMBCT) and showed that both treatments were effective in reducing psychological distress, fear of cancer recurrence and rumination, and improving mindfulness skills, positive mental health and quality of life in a sample of distressed patients with cancer compared with care as usual (CAU; [8]). Despite these positive effects, more patients in the eMBCT condition did not complete the intervention when compared to face-to-face MBCT. Post-treatment interviews revealed that although some participants reported liking the flexibility in where, how, and when to participate in the online version, a proportion of patients were less satisfied with the asynchronicity of the responses and reported lacking peer support during eMBCT [7].

These experiences and barriers to participation in online interventions are known to occur across populations [30]. Although it is still under debate on how optimal support should be given [17], it has been suggested that good quality online interventions consider the heterogeneity in patients characteristics and skills, the context, and evidence from previous development activities [17].

Building on the findings of our BeMind study and considering the developing field of online interventions, we have developed two new versions of the effective eMBCT program with therapist assistance in a co-creation process together with relevant stakeholders. Due to the COVID pandemic, and to provide optimal access for patients, both conditions will be offered fully online. Patients will be randomized to either of the conditions or to CAU. Patients initially randomized to CAU will follow a second randomization. The first condition will be blended eMBCT, combining four online group sessions with a therapist with four individual therapist-assisted online sessions via the E-health platform or app. This will allow patients to have peer support during the group meetings while keeping flexibility for the individual sessions. The second condition will be unguided eMBCT in which patients will follow the program individually, supported by an automated coach via the E-health platform or app. This type of delivery does not depend on a mindfulness teacher at all, making it more accessible to a big group of patients. As adherence to unguided interventions often is lower than intended, persuasive technology known to improve adherence was included, such as reminders, videos, and virtual coaches [15].

The primary objective of our RCT is to evaluate the effectiveness of blended and unguided eMBCT versus CAU in reducing psychological distress in cancer patients and survivors immediately post-intervention. Secondary objectives are to evaluate the interventions’ effects post-intervention on secondary psychological outcome measures, to test the consolidation of treatment effects up to nine months post-treatment, and to evaluate the cost-effectiveness of both eMBCTs. In addition, we will investigate the effect of different moderators on treatment outcome and explore working mechanisms by looking at possible mediators. A better understanding of both moderators and mediators contribute to elucidate how these programs work and for whom. We expect that both blended and unguided eMBCT will be more effective in improving psychological distress and secondary outcomes compared to CAU. Moreover, we expect unguided eMBCT to compare favorably to blended eMBCT in terms of cost-effectiveness.

## Methods

### Study design

**Fig. 1:**
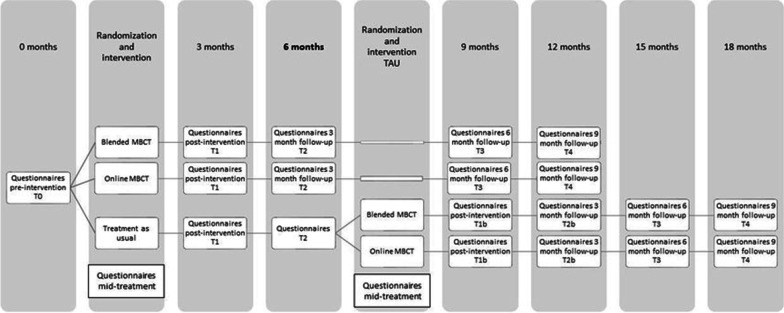
Study design

The current study is a three-armed parallel RCT with blended eMBCT, unguided eMBCT, and CAU. Participants complete the following assessments: baseline (T0), mid-treatment, immediately post-treatment (T1; primary endpoint) and three months later (T2). After T2, participants in CAU will be randomized a second time to blended eMBCT or unguided eMBCT (see Fig. 1). All participants in either blended eMBCT or unguided eMBCT will also receive follow-up questionnaires at six (T3) and nine (T4) months post-treatment.

### Setting

The trial will be conducted by the Radboud university medical center for mindfulness in Nijmegen, The Netherlands. In both intervention conditions, participants will follow the individual sessions through the online web-based platform Minddistrict. Participants will be invited to create a secured personal account, after which they can log in through a website on their computer/laptop or by installing the platform’s app on their tablet or mobile phone. For the blended eMBCT, additional group sessions will be provided through the online video conferencing program Zoom.

### Study population

To be eligible for the study, participants (1) have to be diagnosed with cancer (regardless of the time since diagnosis, type or stage of the disease); (2) have access to internet plus computer literacy; (3) have a good command of the Dutch language; and (4) have to be willing to participate in either intervention condition. Exclusion criteria are: (1) having participated in a mindfulness course before (> four sessions); (2) severe psychiatric comorbidity that warrants acute treatment (psychosis, mania, severe personality disorders, active suicidal ideation); (3) dependence on drugs or alcohol; and (4) severe cognitive impairments.

### Procedure

Participants will be recruited through different online and offline strategies. Offline recruitment will include putting up posters in healthcare settings (e.g., hospitals, cancer support centers) and distributing flyers through health care professionals (such as psychologists, social workers, or nurses). Online recruitment will include online posts placed on websites of cancer-related organizations or patient group fora, by providing webinars or online workshops, and through social media platforms.

Interested participants will be able to enroll themselves by contacting the investigators via email, phone, or by filling the contact form on the Buddy trial website. A member of the research team will then call them to provide information about the study, verify the inclusion criteria and answer any questions they may have. For stratification, participants will be asked if they have or have had breast cancer (vs. other types of cancer), and what is (or has been) the intent of the treatment (curative vs. palliative). Eligible participants will then receive detailed written information about the study and the informed consent form by regular mail and email.

Participants who complete and return the signed consent form will be enrolled in the trial and proceed with the baseline assessments through the secure data management system Castor EDC. After completion of the baseline assessments, participants will be randomized.

Recruitment will be continuously monitored to ensure that the sample size is being reached. If participant enrollment is not as expected, strategies to boost recruitment will be discussed with the research team. In addition, to safeguard completion of the assessments and participant retention in the study, researchers will send reminders through different contact channels based on the participants’ preference (email, phone calls, mail, WhatsApp, text) and also send postcards to thank participants for completing their assessments. Participants can decide to discontinue participation at any time point during the study. Recruitment will take place between January 2021 and September 2023.

### Randomization

Upon the start of the trial, participants were randomized to blended versus unguided versus CAU with a 1:2:1 ratio to one of the three groups because of anticipated higher drop-out rate in the unguided eMBCT. However, upon inspection of completion rates after one year of enrollment, it appeared that the drop-out rates were quite similar across the three conditions. Therefore, the remainder of participants are randomly assigned with a 1:1:1 ratio. Participants randomized to CAU will be randomly allocated to one of the two intervention conditions after six months (T2). We will use permuted blocks of random sizes. Participants will be stratified for breast cancer versus other cancer, and palliative versus curative treatment intent. Randomization will be performed by a member of the research team using Castor EDC, with complete allocation concealment. Participants and trainers cannot be blinded to treatment allocation, and outcome data will involve self-report measures. However, data analysis will be performed by an investigator who has not taken part in contacting participants, randomization, and who will be blinded for results until completion of follow-up assessments.

### Intervention and comparator

The blended and unguided eMBCT training are based on the MBCT program developed by Segal et al. [25]. However, in co-creation with cancer patient representatives and eHealth experts, we adapted the program to the needs of the target population. For instance, we included psychoeducation about cancer and mindfulness (session one), and grief (sessions four and five). See Table 1 for the content of the interventions. We developed the online program in the eHealth platform Minddistrict to be appealing by using persuasive technology, such as reminders and video fragments of peers who had participated in eMBCT before. After development, we pilot-tested both formats (blended and unguided eMBCT) to evaluate the feasibility and acceptability of the program and patients’ satisfaction, experiences, and suggestions for improvement.

**Table 1:** Intervention content

Theme of the session	Content of the session	Meditation exercise	Homework	Modality for blended eMBCT*
0. Introduction to MBCT	What the online mindfulness training looks like	None	None	Individual
	What the online app looks like and how to use it			
	What can mindfulness do for you			
1. The automatic pilot	Intention of participation	Body scan	Body scan	Group
	Raisin exercise		Mindful eating	
	Psycho-education: how mindfulness can help for physical and mental problems after cancer		Daily mindful activity	
2. Thoughts are not facts	Observation exercises: “looking out the window” and “walking down the street”	Body scan	Body scan	Individual
Seeing clearly		Sitting meditation: instructions and attention to breathing	Attention to the breathing 5–10 min daily	
			Daily mindful activity	
			Complete diary positive experiences	
3. From doing to being	Triangle of awareness	Movement exercises	-Alternating sitting meditation and movement exercises	Group
	Reflect on positive experiences	Sitting meditation: breathing and body-awareness	Three minutes breathing space at fixed times	
			Daily mindful activity	
			Complete diary negative experiences	
4. Stay with it and allow what is	Reflect on negative experiences	Sitting meditation	-Alternating between sitting meditation, movement exercises, or walking meditation	Individual
Staying present	Psycho-education: Grief process	Walking meditation	Three minutes breathing space and apply it during stressful moments	
		Introduction to Three minutes breathing space	Complete stress diary	
5. Finding/creating space to make choices Or: Responding to stress instead of reacting	-Reflect on how to respond to stress. Reaction vs response	Sitting meditation with a difficulty	Alternating between sitting meditation, or movement exercises	Group
	Psycho-education: phases and tasks to grief	Three minutes breathing space: use as a coping mechanism	Three minutes breathing space once a day and during difficult situations	
	Halfway reflection of the training	Movement exercises	Paying attention to reactions during stressful situations and practice giving a response	
			Complete difficult communication	
6. Mindful communication	Mindful communication	Movement exercises standing up	Alternating between sitting meditation, lying, or standing movement meditation, or body scan without audio	Individual
Communication with awareness	Energy givers and energy takers	Three minutes breathing space: use as a coping mechanism	Three minutes breathing space	
			Mindful communication	
			Energy balance and action plan	
Silent day	Follow the silent day with the app	Body scan		Individual
		Movement meditation		
		Sitting meditation		
		Loving kindness meditation		
7. Taking care of yourself	Create an action plan	Movement exercise without guidance	Mindful exercise of personal choice	Individual
		Sitting meditation without guidance	Three minutes breathing space	
		Three minutes breathing space	Discuss the action plan with a loved one	
			Self-evaluation	
8. The 8th week continues for the rest of your life	Reflection on the course	Body scan		Group
	How to continue practicing mindfulness in your daily life	Sitting meditation (5 words to remember mindfulness)		

^*^For unguided eMBCT all sessions are individual

For both treatment conditions, the program sessions will include mindfulness meditation exercises, psychoeducation, and reflections. In addition to the MBCT sessions, participants will be asked to do daily home practice of 30–45 min a day. Participants can access all necessary information, e.g., audio files of exercises, and recording forms of assignments around the theme of the session through a personal, secure webpage. In both treatment conditions, participants will be allowed to join the program with a significant other.

Blended eMBCT will consist of four 2.5-h group sessions through Zoom (sessions one, three, five, and eight), led by a mindfulness teacher. Participants will complete the other sessions individually through the online platform. Mindfulness teachers will provide online feedback to participants for these sessions. The mindfulness teachers involved in the project, will be health care professionals experienced in psycho-oncology who meet the qualification criteria of the Association of Mindfulness Teachers based in The Netherlands and Flanders, which is in line with the UK Network for Mindfulness-Based teachers’ criteria (2015). In addition, teachers will have regular peer discussion sessions supervised by a senior mindfulness teacher.

Participants in the unguided eMBCT condition will be provided with the whole intervention through the Minddistrict platform. They will receive weekly access to one of the online mindfulness sessions, which involve the same themes, exercises, and homework as those in the blended eMBCT. However, there is no involvement from a mindfulness teacher. An online coach in the program provides automated support and feedback throughout the sessions.

Participants assigned to CAU will receive notices and reminders to complete trial measures. They will be contacted with information on intervention groups after T2.

Participants in any of the three conditions, can have any form of medical, psychological or paramedical care during the study period they require, except for MBIs outside of the study context. Participants may choose to discontinue the intervention and study at any time. We do not envision the need to modify the intervention for any participant or to discontinue their participation. However, any relevant information disclosed to the teachers or members of the research team that are a cause of concern will be discussed with the team supervisor and trial leadership. Pertinent recommendations for obtaining appropriate support will be made to the participant, and if needed trial participation may be ended.

### Outcome measures

#### Primary outcome measure

The primary outcome measure of the study will be psychological distress at post-treatment (T1), assessed with the total scores from the Hospital Anxiety and Depression Scale (HADS). The HADS is a 14-item self-report questionnaire that has been widely used to measure psychological distress in cancer patients [21]. Items are scored on a zero-three scale and are summed to obtain a total distress score (range 0–42). Higher scores represent more distress.

#### Secondary outcome measures

**Fig. 2:**
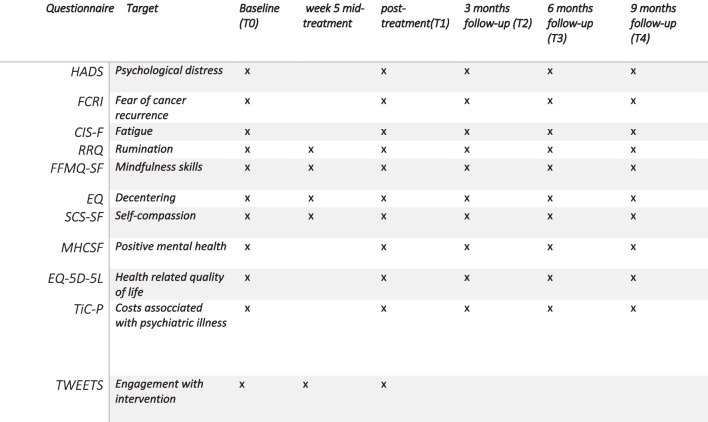
Measurement scheme

Secondary outcomes include multiple psychological outcomes, fatigue, mindfulness skills and mediating variables as well as cost and engagement with the interventions. Below is a brief description of each questionnaire (see Fig. 2 for an overview of the outcome measures at each assessment).

*Fear of cancer recurrence* will be assessed using the nine-item Fear of Cancer Recurrence Inventory-severity subscale (FCRI-SF) [26]. Each item assesses fear of cancer recurrence on a five-point Likert scale ranging from zero to four. Higher scores indicate higher levels of FCR. The Dutch version has an overall Cronbach’s alpha of 0.93 [13].

*Fatigue* will be measured using the Fatigue Severity Subscale of the Checklist Individual Strength (CIS). The eight-item CIS-Fatigue Severity subscale measures the patient's fatigue levels over the past two weeks on a seven-point Likert scale, with higher scores indicating higher levels of fatigue. The Fatigue Severity subscale from the CIS has shown to have a Cronbach’s alpha of 0.94 in a Dutch sample [31].

*Rumination* will be assessed using the 12-item rumination subscale of the Rumination and Reflection Questionnaire (RRQ). Items are scored on a five-point Likert scale, with higher scores indicating more rumination. The Dutch version has shown to have a good reliability with Cronbach’s alphas ranging between 0.88 and 0.92 [20].

*Mindfulness skills* will be measured using the Five Facet Mindfulness Questionnaire Short Form (FFMQ-SF). The 24-item FFMQ-SF consists of five subscales: observing, describing, acting with awareness, non-judging of inner experience, and non-reactivity to inner experience. Items are scored on a five-point Likert scale. Higher scores are indicative of someone who is more mindful in their everyday life. The FFMQ-SF validated in a Dutch sample showed alpha coefficients for all subscales ranging between 0.73 and 0.91 [1].

*Decentering* will be assessed with the decentering subscale of the Experiences Questionnaire. The 11-item Decentering subscale of the Experiences Questionnaire (EQ) [11] measures the ability to observe one's thoughts and feelings as temporary, objective events in the mind. Participants rate items on a five-point Likert scale. Higher scores indicate greater ability to decenter. Currently, a validated Dutch version of this questionnaire is not available. We have translated the English questionnaire to Dutch following standard forward–backward translation procedures.

*Self-compassion* will be evaluated using the 12-item Self-Compassion Scale Short Form (SCS-SF), which measures the capacity for self-compassion. Items are scored on a five-point Likert scale. Higher scores reflect higher levels of self-compassion. Items from the SCS-SF are divided across six subscales: self-kindness, self-judgement, common-humanity, isolation, mindfulness, and over identification. The Dutch version of the SCS–SF has been shown to have Cronbach's alpha ≥ 0.86 [24].

*Positive mental health* will be assessed using Mental Health Continuum-Short Form (MHC-SF). The 14-item MHC-SF measures positive mental health, consisting of three subscales: emotional well-being, psychological well-being, and social well-being. Items assess the frequency with which respondents experience each symptom of positive mental health on a six-point Likert scale ranging from zero to five, for the past month. Higher scores indicate greater levels of positive well-being. The MHCSF has been shown to have a Cronbach’s alpha of 0.89 in a Dutch sample [18].

To measure *health related quality of life* of cancer patients, we will use the EuroQol-5D-5L (EQ-5D) [29]. The EQ-5D is a generic instrument comprising five domains: mobility, self-care, usual activities, pain/discomfort, and anxiety/depression. The EQ-5D index is obtained by applying predetermined weights to the five domains. This index gives a societal-based global utility score of the participant's health status on a scale with zero (death) and one (perfect health).

*Costs associated with Psychiatric illness* will be assessed using the Trimbos/iMTA questionnaire for Costs associated with Psychiatric illness (TiC-P) [2]. The TiC-P collects information on direct healthcare use (e.g., general practitioner, mental healthcare, medication, and hospital day care visits) and paid and informal work-related productivity losses. We will use a retrospective three-month period for determining healthcare and societal costs.

*Engagement with the intervention* will be assessed with the Twente Engagement with Ehealth Technologies Scale (TWEETS). The TWEETS consists of nine items equally divided over three subscales: behavioral engagement, cognitive engagement, and affective engagement. Participants indicate on a scale of one to five to which extent they agree with different statements. Cronbach alpha of the TWEETS has shown to be 0.86 in a Dutch sample [16].

### Data management

Data, including outcome measures, for the Buddy trial will be collected using Castor EDC. Once all outcome data is collected, data will be exported to the statistics software program R. Checking and cleaning of the data will occur within R and be done by members of the study team. All data will be kept and stored according to the Personal Data Protection Act. Data collected throughout the study will be stored using encrypted digital files within password protected folders with limited access to a restricted number of researchers. Participants will be assigned a unique participant identification number upon inclusion to ensure confidentiality. This number and the corresponding personal information are only accessible to the principal investigator and team members under their supervision. Contact details will be stored separately from all the other research data collected during the study. All data will be securely stored for 15 years after the study is completed. During the trial, access to the data will be limited to the study investigators. Once trial results are reported, de-identified data will be made available upon reasonable request.

It is estimated that the risk of harm is not increased when participating in this study. With regard to NFU-classification the risk is negligible. Therefore a data monitoring committee is not needed for this study.

### Safety reporting

In accordance with the WMO, the sponsor will suspend the study if there is sufficient ground that continuation of the study will jeopardize subject health or safety. Adverse events that may occur during the study will be examined at each assessment by asking people if they noticed any unpleasant effect that appear to be causally related to the MBCT training. Any (serious) adverse event reported by the subject or investigator, or team will be noted and reported to the competent authority.

## Analysis

### Power calculation and sample size

Power analysis is based on a three-group ANCOVA, with psychological distress post-treatment as primary endpoint, corrected for baseline psychological distress. We will use a close testing procedure for pairwise comparisons between blended eMBCT vs. CAU, and unguided eMBCT vs. CAU. In our previous study, we found that the effect size of group MBCT was d = 0.45 and that of therapist-assisted eMBCT was d = 0.71 [8]. So, we expect the effect size of blended eMBCT to be d = 0.58 (the mean of the effect sizes of the group and therapist-assisted eMBCT in our previous trial). We estimate the effect size of the unguided eMBCT vs CAU to be d = 0.45 (the smallest effect found in our previous study). Alpha is set at 0.05 and the Pearson correlation between T0 and T1 at 0.50 (based on our previous study). To ensure a power of 0.80 in the pairwise comparison eMBCT vs CAU, a minimal group of 65 for CAU is required. This results in a power of 0.95 of the three-group ANCOVA, and a power of 0.92 for the comparison eMBCT vs CAU and 0.82 for the comparison blended eMBCT vs CAU.

Based on our previous study, we anticipate 20% dropout in blended eMBCT, 40% dropout in unguided eMBCT, and 0% in CAU, which rendered a target sample size of at least 254 participants: 81 in blended eMBCT, 108 in unguided eMBCT, and 65 in CAU. However, after inclusion of the first 80 participants, drop-out appeared to be similar across the three conditions (28%). We therefore adjusted our target to include at least 86 participants in each arm.

### Statistical analysis

Descriptive statistics will be used to describe participant characteristics, including demographic and clinical variables at baseline.

Analyses will be conducted by a statistician blind to trial arm allocation. All primary statistical analyses use an intention to treat (ITT) approach with an alpha level of 0.05 to estimate score differences between intervention and care as usual participants. To minimize the possibility of bias from missing outcome data, we will use multiple imputation by chained equations to generate 20 imputed datasets, using 15 cycles per imputed dataset.

The primary outcome analysis will compare HADS total scores between patients in the blended eMCBT and CAU arms and between the unguided eMCBT and CAU arms at post-treatment (T1). We will use a closed testing procedure with ANCOVA. Group (blended eMBCT, unguided eMBCT, and CAU) will be added as between subject factor, and baseline HADS scores and stratification variables (breast cancer vs. other and curative vs. palliative treatment intent) will be added as covariates. In case of an overall significant test, we will conduct pairwise comparisons between blended eMBCT vs. CAU, and unguided eMBCT vs. CAU. Analyses of secondary outcomes at post-treatment (T1) will be conducted using a similar procedure.

In addition, we will evaluate treatment consolidation effects after blended and unguided eMBCT at follow-up. For each outcome separately, we will perform linear mixed regression analysis. Dependent variables will be the primary outcome or secondary outcomes at T1, T2, T3, and T4. Independent variables will be time, intervention (blended or unguided eMBCT), their interaction, and the baseline level (T0) of the outcome.

Exploratory moderation analyses of the primary outcome at T1 will be performed by including a three-way interaction term among condition, time, and possible moderators: sex, age, cancer diagnosis, anticancer treatment intent, time since diagnosis, rumination, self-compassion, mindfulness, and psychological distress. First, moderators will be assessed in two separate analyses of either intervention compared with CAU. Second, moderators will be assessed in analyses of the two intervention conditions only. Explorative moderation analyses of the primary outcome during long-term follow-up will be performed by using linear regression with psychological distress at T1, T2, T3, and T4 as dependent variable.

Moreover, the mid-treatment assessments will be used to examine whether the change of possible mediators (rumination, mindfulness, decentering, self-compassion, and engagement with the intervention) over the first half of training is predictive of the change of psychological distress over the full duration of the training. For the mediation analyses, we will follow the recommendations of Preacher and Hayes [23] for multiple mediation models. In all mediation analyses, severity of psychological distress at T1 will be controlled for baseline levels.

Economic evaluation will be based on the general principle of a cost-utility analysis which is performed alongside the RCT. We will compare the three conditions: blended eMBCT, unguided eMBCT, and CAU. Primary outcome measures for economic evaluation will be costs (we will follow Dutch guidelines for costs research and quality adjusted life years measured by EQ-5D. The societal costs will be operationalized by including productivity losses/gains applying the friction cost method on a per patient basis by using the TiC-P. The incremental cost-effectiveness ratio “cost per Quality-Adjusted Life Year gained (QALY)” will be based on EQ-5D utilities, and uncertainty surrounding these parameters will be determined using the bootstrap method. A cost-acceptability curve will be derived that will be able to evaluate efficiency by using a range of thresholds (willingness to pay for QALY gained). The impact of uncertainty surrounding relevant deterministic parameters on the ICER will be subsequently explored using one-way sensitivity analyses on the range of extremes.

## Discussion

The number of cancer patients and survivors is increasing [9], and a significant proportion experience severe psychological distress [32]. MBCT, including when delivered online, has shown to reduce psychological distress for cancer patients and survivors [8]. It remains to be elucidated, however, which online delivery format(s) provide an optimal balance between user flexibility, patient engagement, and effectiveness.

In our study, we will investigate different formats of eMBCT delivery, as well as explore which type of treatment-blended or unguided eMBCT-works for whom. By evaluating different online formats, we strive to be able to broadly disseminate effective, easily accessible, sustainable, and relatively low-cost MBCT to people with cancer.

## Data Availability

The datasets used and/or analyzed during the current study will become available from the corresponding author on reasonable request after the trial is completed. Results will be disseminated in relevant peer-reviewed journals and scientific conferences.

## Appendix

Table 1 and Figs. 1 and 2.

## Abbreviations

CAU: Care as usual
CIS: Checklist Individual Strength-Fatigue
EQ: Decentering Experiences Questionnaire
ES: Effect size
EQ-5D: EuroQol-5D-5L
eMBCT: Internet mindfulness based therapy
FCRI: Fear of Cancer Recurrence Inventory
FFMQ-SF: Five Facet Mindfulness Questionnaire Short Form
HADS: Hospital Anxiety and Depression Scale
ITT: Intention to treat
KWF: Dutch Cancer Society
MBI: Mindfulness based interventions
MCBT: Mindfulness based cognitive therapy
MBSR: Mindfulness based stress reduction
METC: Medical research ethics committee (MREC), in Dutch: medisch-ethische toetsingscommissie (METC)
MHCSF: Mental Health Continuum Short Form
NCCN: National Comprehensive Cancer Network
NMB: Net monetary benefit
RRQ: Rumination and Reflection Questionnaire
SCS-SF: Self-Compassion Scale Short Form
TiC-P: Costs Associated with Psychiatric Illness
TWEETS: Twente Engagement with E-Health Technologies
WMO: Medical Research Involving Human Subjects Act

## References

[1] Bohlmeijer E, ten Klooster PM, Fledderus M, Veehof M, Baer R (2011). Psychometric Properties of the Five Facet Mindfulness Questionnaire in depressed adults and development of a short form. Assessment.

[2] Bouwmans C, De Jong K, Timman R, Zijlstra-Vlasveld M, Van der Feltz-Cornelis C, Tan SS (2013). Feasibility, reliability and validity of a questionnaire on healthcare consumption and productivity loss in patients with a psychiatric disorder (TIC-P). BMC Health Serv Res.

[3] Brebach R, Sharpe L, Costa DSJ, Rhodes P, Butow P (2016). Psychological intervention targeting distress for cancer patients: a meta-analytic study investigating uptake and adherence. Psychooncology.

[4] Bruggeman Everts FZ, van der Lee ML, de Jager ME (2015). Web-based individual mindfulness-based cognitive therapy for cancer-related fatigue—a pilot study. Internet Interv.

[5] Cillessen L, Johannsen M, Speckens AE, Zachariae R (2019). Mindfulness-based interventions for psychological and physical health outcomes in cancer patients and survivors: a systematic review and meta-analysis of randomized controlled trials. Psychooncology.

[6] Cho D, Park C (2018). Barriers to physical activity and healthy diet among breast cancer survivors: a multilevel perspective. Eur J Cancer Care.

[7] Compen FR, Bisseling EM, Schellekens MP, Jansen ET, van der Lee ML, Speckens AE (2017). Mindfulness-based cognitive therapy for cancer patients delivered via internet: qualitative study of patient and therapist barriers and facilitators. J Med Internet Res.

[8] Compen F, Bisseling E, Schellekens M, Donders R, Carlson L, van der Lee M (2018). Face-to-face and internet-based mindfulness-based cognitive therapy compared with treatment as usual in reducing psychological distress in patients with cancer: a multicenter randomized controlled trial. JCO.

[9] Cancer survival statistics: World cancer research fund international [Internet]. WCRF International. 2022 [cited 2022Oct6]. https://www.wcrf.org/cancer-trends/cancer-survival-statistics/.

[10] Fawzy FI (1995). Critical review of psychosocial interventions in cancer care. Arch Gen Psychiatry.

[11] Fresco DM, Moore MT, van Dulmen MH, Segal ZV, Ma SH, Teasdale JD (2007). Initial psychometric properties of the experiences questionnaire: validation of a self-report measure of decentering. Behav Ther.

[12] Goldberg SB, Riordan KM, Sun S, Davidson RJ (2022). The empirical status of mindfulness-based interventions: a systematic review of 44 meta-analyses of randomized controlled trials. Perspect Psychol Sci.

[13] van Helmondt SJ, van der Lee ML, de Vries J (2017). Translation and validation of the Dutch version of the Fear of Cancer Recurrence Inventory (FCRI-NL). J Psychosom Res.

[14] Kabat-Zinn J (1982). An outpatient program in behavioral medicine for chronic pain patients based on the practice of mindfulness meditation: theoretical considerations and preliminary results. Gen Hosp Psychiatry.

[15] Kelders SM, Kok RN, Ossebaard HC, Van Gemert-Pijnen JEWC (2012). Persuasive system design does matter: a systematic review of adherence to web-based interventions. J Med Internet Res.

[16] Kelders SM, Kip H, Greeff J (2020). Psychometric evaluation of the Twente Engagement with Ehealth Technologies Scale (TWEETS): evaluation study. J Med Internet Res.

[17] Kip H, Keizer J, da Silva MC, Beerlage-de Jong N, Köhle N, Kelders SM (2022). Methods for human-centered eHealth development: narrative scoping review. J Med Internet Res.

[18] Lamers SM, Westerhof GJ, Bohlmeijer ET, ten Klooster PM, Keyes CL (2011). Evaluating the psychometric properties of the Mental Health Continuum-Short Form (MHC-SF). J Clin Psychol.

[19] Lengacher CA, Reich RR, Ramesar S, Alinat CB, Moscoso M, Cousin L (2018). Feasibility of the mobile mindfulness-based stress reduction for breast cancer (mMBSR(BC)) program for symptom improvement among breast cancer survivors. Psychooncology.

[20] Luyckx K, Schwartz SJ, Berzonsky MD, Soenens B, Vansteenkiste M, Smits I (2008). Capturing ruminative exploration: extending the four-dimensional model of identity formation in late adolescence. J Res Pers.

[21] Mitchell AJ, Meader N, Symonds P (2010). Diagnostic validity of the Hospital Anxiety and Depression Scale (HADS) in cancer and palliative settings: a meta-analysis. J Affect Disord.

[22] Niedzwiedz CL, Knifton L, Robb KA, Katikireddi SV, Smith DJ (2019). Depression and anxiety among people living with and beyond cancer: a growing clinical and research priority. BMC Cancer.

[23] Preacher KJ, Hayes AF (2008). Asymptotic and resampling strategies for assessing and comparing indirect effects in multiple mediator models. Behav Res Methods.

[24] Raes F, Pommier E, Neff KD, Van Gucht D (2011). Construction and factorial validation of a short form of the Self-Compassion Scale. Clin Psychol Psychother.

[25] Segal Z, Williams M, Teasdale J (2018). Mindfulness-based cognitive therapy for depression.

[26] Smith AB, Costa D, Galica J, Lebel S, Tauber N, van Helmondt SJ (2020). Spotlight on the Fear of Cancer Recurrence Inventory (FCRI). PRBM.

[27] Sung H, Ferlay J, Siegel RL, Laversanne M, Soerjomataram I, Jemal A (2021). Global cancer statistics 2020: GLOBOCAN estimates of incidence and mortality worldwide for 36 cancers in 185 countries. CA A Cancer J Clin.

[28] Thewes B, Butow P, Zachariae R, Christensen S, Simard S, Gotay C (2012). Fear of cancer recurrence: a systematic literature review of self-report measures. Psychooncology.

[29] Versteegh M, Vermeulen K, Evers MAA S, de Wit GA, Prenger R, Stolk AE. Dutch tariff for the five-level version of EQ-5D. Value Health. 2016;19(4):343–52. 10.1016/j.jval.2016.01.003 27325326

[30] Webb TL, Joseph J, Yardley L, Michie S (2010). Using the internet to promote health behavior change: a systematic review and meta-analysis of the impact of theoretical basis, use of behavior change techniques, and mode of delivery on efficacy. J Med Internet Res.

[31] Worm-Smeitink M, Gielissen M, Bloot L, van Laarhoven H, van Engelen B, van Riel P (2017). The assessment of fatigue: psychometric qualities and norms for the Checklist Individual Strength. J Psychosom Res.

[32] Zabora J, BrintzenhofeSzoc K, Curbow B, Hooker C, Piantadosi S (2001). The prevalence of psychological distress by cancer site. Psychooncology.

[33] Zernicke KA, Campbell TS, Speca M, Ruff KM, Flowers S, Tamagawa R (2016). The eCALM Trial: eTherapy for cancer applying mindfulness. Exploratory analyses of the associations between online mindfulness-based cancer recovery participation and changes in mood, stress symptoms, mindfulness, posttraumatic growth, and spirituality. Mindfulness.

