# Correction to: effect of blended and unguided online delivery of mindfulness-based cognitive therapy versus care as usual on distress among cancer patients and survivors: protocol for the three-arm parallel randomized controlled Buddy trial

**DOI:** 10.1186/s40359-023-01083-9

**Published:** 2023-02-13

**Authors:** Nasim Badaghi, Mette van Kruijsbergen, Judith Prins, Saskia Kelders, Linda Cillessen, Félix Compen, Rogier Donders, Linda Kwakkenbos, Anne Speckens

**Affiliations:** 1 Department of Psychiatry, Radboud University Medical Center, 966, Postbus 9101, 6500 HB Nijmegen, The Netherlands; 2 Department of Medical Psychology, Radboud University Medical Center, Nijmegen, The Netherlands; 3 Department of Psychology, Health, and Technology, University of Twente, Enschede, The Netherlands; 4 Optentia Research Unit, North-West University, Potchefstroom, South Africa; 5 Radboud Institute for Health Evidence, Radboud University Medical Center, Nijmegen, The Netherlands; 6 Behavioural Science Institute, Clinical Psychology, Radboud University Nijmegen, Nijmegen, The Netherlands

BMC Psychology (2023) 11:21

10.1186/s40359-023-01052-2

Following publication of the original article [1], the authors flagged that a 'model consent form’ completed in the Dutch language had been erroneously added to Figure 2. The figure has since been corrected to remove the form. The authors thank you for reading this correction and apologize for any inconvenience caused.

